# Point-of-use water filters: do they provide sterile water over the entire time of the manufacturer’s warranty?

**DOI:** 10.1186/2047-2994-4-S1-P53

**Published:** 2015-06-16

**Authors:** M Dangel, A Widmer

**Affiliations:** 1 Division of Infectious Diseases & Hospital Epidemiology, University Hospital Basel, Basel, Switzerland

## Introduction

Hospital water supplies may contain waterborne pathogens: *P. aeruginosa* has been implicated in transmission of healthcare-associated infections (HAIs), in particular in ICUs. Point-of-use (POU) water filters deliver sterile water providing a service life between 7 and 62 days. Until recently, this time was limited to 30 days, and was extended during the last years. However, no independent investigation proved these filters to be able to provide sterile water for the entire lifetime claimed by the manufacturer.

## Objectives

First, validation of the manufacturer’s claimed standing time to provide sterile water, and secondly, to evaluate the filter’s performance after the expiration of its service life.

## Methods

Two POU water filters are installed in 2 rooms (R1, R2) on a ward to test the risk of contamination by tap water. Two types of filters were tested during a 7 months period:

Pall Q-Point (service life by company: 62 days) and Anios 31DA+ (service life according to company: 31 days). Samples were collected at the same date in two adjacent rooms on the same ward as control (C1, C2) that have the same main water supply. 36 samples (100ml each) were taken from faucets with filters and 23 from control without filters and analyzed for total viable count per millilitre, and number of bacteria and *Pseudomonasaeruginosa* (PSAE) per 100 millilitres.

## Results

Microbiologic examinations of tap water revealed a growth of PSAE in the first sample collected after 30 days in R1 (Filter nr. 1/10). In R2 the first filter was changed after 56 days (became clogged, delivered sterile water during 56 days). One PSAE colony appeared in R2 much later, after 203 days (Filter nr. 4/5).

total viable count per millilitre mean (SD)

[R1+R2] 2,58 (17,1); [C1+C2] 122,5 (205,0); p < 0,001

number of bacteria per 100ml mean (SD)

[R1+R2] 12,7 (32,1); [C1+C2] 104,17 (32,2);p < 0,001

*Pseudomonas aeruginosa* per 100ml mean (SD)

[R1+R2] 6,29 (22,7); [C1+C2] 1,09 (5,4); *p* = 0,13

Molecular typing of the strains demonstrated high similarity suggesting that this PSAE strain is endemic to the water supply of our hospital.

## Conclusion

POU water filters significantly reduce the microbiological burden of tap water. This study confirms service lives of the filters: using filters beyond the manufacturer’s recommendation may lead to high-level contamination with *Pseudomonas aeruginosa*.

## Disclosure of interest

None declared.

